# Acute Transverse Myelitis in Pregnancy: The Use of ProSeal Laryngeal Mask Airway Without Curarization for Emergency Cesarean Section

**DOI:** 10.7759/cureus.26185

**Published:** 2022-06-21

**Authors:** Shivam Shekhar, Sakshi Kadian, Sony Sony, Sujata Chaudhary, Jubin Jakhar

**Affiliations:** 1 Department of Anesthesiology, All India Institute of Medical Sciences, Rishikesh, IND; 2 Anesthesiology, Guru Teg Bahadur (GTB) Hospital, New Delhi, IND; 3 Anesthesiology, Vardhman Mahavir Medical College (VMMC) and Safdarjung Hospital (SJH), New Delhi, IND

**Keywords:** general anesthesia, curarization, lower limb weakness, inflammation, cesarean section, proseal laryngeal mask airway, transverse myelitis

## Abstract

Transverse myelitis is a rare inflammatory neurological disorder of the spinal cord that damages the myelin covering the spinal cord and thus produces sensory, motor, and autonomic symptoms. A 26-year-old primigravida of 40 weeks gestation presented to the obstetric emergency of our hospital with complaints of weakness in both lower limbs and inability to walk for four days. A diagnosis of acute transverse myelitis was made, and due to fetal distress and arrest of labor in the second stage, an emergency cesarean section was planned. Considering the risks associated with the neuraxial technique and muscle relaxants, cesarean section was planned under general anesthesia and was successfully done with ProSeal laryngeal mask airway (LMA) using propofol and sevoflurane without muscle relaxant.

## Introduction

Transverse myelitis is a rare neurological disorder that damages the myelin covering the spinal cord, usually symmetrical in nature. This focal spinal cord inflammation can cause sensory problems, muscle weakness, paralysis, pain, autonomic dysfunction, or bladder and bowel dysfunction. Anesthetic management of such patients is a big challenge. This case report is about the successful anesthetic management of a term pregnant lady with bilateral lower limb paralysis who underwent an emergency cesarean section.

## Case presentation

A 26-year-old primigravida was admitted to the obstetric and gynecology department at 40 weeks gestation with complaints of weakness in both lower limbs and inability to walk for four days. She was unable to sit without support. On neurological examination, she had graded sensory loss below T6 (sixth thoracic) dermatomal distribution. No zone of paresthesia or hyperesthesia was found. Her lower limbs were flaccid, while bladder and bowel function was intact. She weighed 66 kg and had a height of 158 cm, leading to a body mass index (BMI) of 26.4 kg/m^2^. Her hemodynamics were stable with blood pressure (BP) of 126/78 mmHg and heart rate (HR) of 66/minute. Routine blood investigation showed hemoglobin of 12.4 g/dL, hematocrit of 38%, platelet count of 2.6 lakh/mm^3^, total leukocyte count of 12,000/mm^3^, serum sodium level of 134 mEq/L, serum potassium level of 3.8 mEq/L, blood urea of 24 mg/dL, and serum creatinine of 0.9 mg/dL.

**Figure 1:**
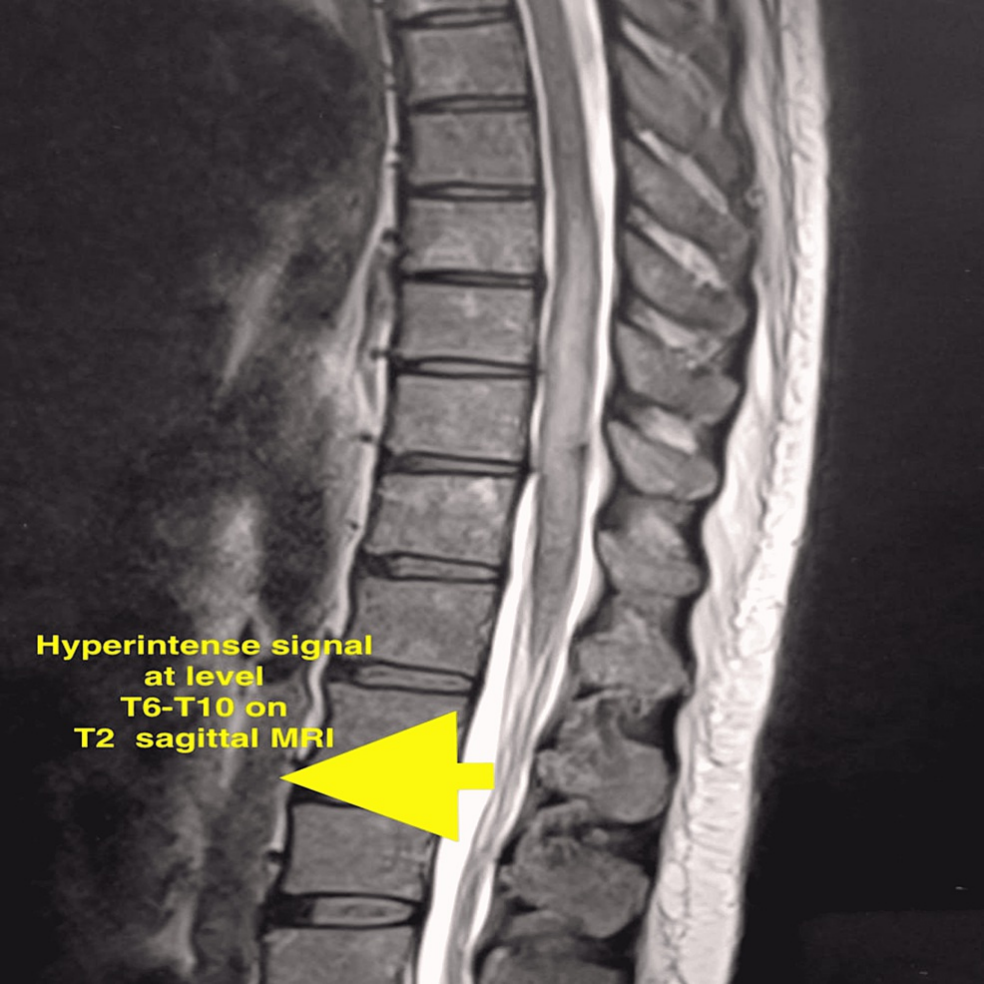
T2 sagittal MRI image of the spine showing hyperintense signal at the T6-T10 levels (arrow)

Neurology consultation was obtained. On neurological examination, muscle power was 5/5 in both upper limbs and 0/5 in both lower limbs. The bulk of both lower limbs was normal, while the tone was decreased in both lower limbs. Deep tendon reflexes showed hyperactive reflex without clonus with the knee, while all other deep tendon reflexes showed a normal response. Bilateral abdominal reflexes were present. Plantar reflex was extensor in nature. All further neurological examinations were normal, including higher mental function, cranial nerve examination, and cerebellar function. No evidence of autonomic dysfunction was found. Based on the above findings, a contrast-enhanced MRI was advised, and a clinical diagnosis of acute transverse myelitis was confirmed by hyperintensity on spinal MRI at the T6-T10 levels (Figure 1).

She was started on injection of methylprednisolone 1 g intravenously that was planned for five days. On the second day, the patient went into labor, but there was an arrest of labor in the second stage. The obstetrician decided to proceed with an emergency cesarean section, considering thick meconium-stained liquor consistent with fetal distress. A pre-anesthetic checkup was done in the preoperative area, and adequate fasting hours were confirmed. On airway examination, her mouth opening was more than three fingers, and Mallampati's grade was 2. On neurological evaluation, graded sensory loss below the T6 level was confirmed. Considering the provisional diagnosis of transverse myelitis, general anesthesia was decided as the anesthesia plan. The risk of anesthesia related to the provisional diagnosis was explained, and written informed consent was taken. Premedication with 10 mg of metoclopramide injection and 40 mg of pantoprazole injection was given intravenously through the in situ 20-gauge cannula. She was shifted to the operation theater, and standard monitoring was established with noninvasive blood pressure, electrocardiography, and pulse oximetry. All emergency drugs were kept ready to control any autonomic hyperreflexia event.

After taking the baseline readings of blood pressure and heart rate and noting baseline electrocardiography rhythm, she was asked to take eight vital capacity breaths in 100% oxygen in one minute. At the end of preoxygenation, 100 mg of propofol was given intravenously. After the loss of verbal response and jaw tone, a ProSeal laryngeal mask airway (LMA) of size four was inserted, and after confirming the correct position, it was taped to the facial skin. About 250 mL of Ringer's lactate was infused during the induction of anesthesia. A suction catheter was advanced into the port for gastric suctioning, and gastric content was suctioned, almost nil in amount. After fixing the ProSeal LMA, the fraction of inspired oxygen was reduced to 50%, and sevoflurane (up to one minimum alveolar concentration) and N2O (50%) were started. Fifteen degrees of left lateral tilt was maintained throughout the procedure using a wedge under the right hip.

A Pfannenstiel incision was made, and surgery was started. No significant change in hemodynamics was observed during the incision. Surgery progressed without difficulty, and a newborn was delivered and handed over to a pediatrician who noted the APGAR score to be 9 at one and five minutes. After the baby's delivery, 3 IU of oxytocin was given as an intravenous bolus over one minute, and 15 IU were given as an infusion. Injection of fentanyl 50 mcg intravenously reduced pain response and avoided autonomic hyperreflexia. Injection methylprednisolone 1 g was given intravenously in view of transverse myelitis. The surgery was completed in less than an hour without any difficulty. No muscle relaxant was used during the surgery, neither at induction nor during the maintenance of general anesthesia, and the patient was ventilating spontaneously throughout the surgery. Bispectral index (BIS) was used to monitor the depth of anesthesia, and its value was maintained between 40 and 60 throughout the perioperative period with intermittent propofol boluses and inhalational anesthetics. Twenty minutes before expected skin suturing, 6 mg of injection ondansetron was given. After surgery, N2O and sevoflurane were stopped, and a fraction of inspired oxygen was increased to 100%. When the patient responded to verbal commands and opened her eyes, the ProSeal LMA was removed after suctioning the gastric port.

During the surgery, blood loss was estimated to be around 600 mL against the maximum allowable blood loss of 830 mL, replaced by 2,000 mL of crystalloid. The vital parameters of the patient were stable throughout the surgery, and no significant hemodynamic change was noted. The patient was then shifted to the Post-Anesthesia Care Unit (PACU) for monitoring. The patient's stay in PACU was also uneventful, and after two hours, she was shifted to the ward for further monitoring and care. The neurological status in the postoperative period was similar to the preoperative level.

## Discussion

Transverse myelitis is a heterogeneous pathological syndrome in which acute or subacute spinal cord inflammation may result in sensory, motor, and autoimmune (bladder, bowel, and sexual) dysfunction below the lesion level [1,2]. It generally shows a bimodal peak in incidence, with most cases occurring in the second and fourth decades of life. It has various etiologies, including infectious, paraneoplastic, drug-induced, autoimmune, and demyelinating diseases. In many patients, etiology remains unknown and is therefore referred to as idiopathic [1,3,4]. Neurological deficits of acute and subacute transverse myelitis typically reach a nadir within weeks. An evolving cause of disease where the deficits keep worsening beyond four weeks is against the diagnosis of transverse myelitis [1]. The presentation and progression vary between individuals. Almost one-third of patients recover well, while the rest of the two-thirds are left with a moderate to severe disability [5].

Sensory symptoms are prevalent in the disease, manifesting as burning, tingling, numbness, and allodynia. Acutely, it may present with spinal shock with hypotension. Later stages of the disease may display signs of autoimmune dysreflexia, including exaggerated hypertension, reflex bradycardia, and arrhythmias. Emergency drugs to control autonomic dysreflexia should be kept intraoperatively and postoperatively [1,4,5]. MRI of the spinal cord and cerebrospinal fluid analysis (cell count, proteins, glucose, oligoclonal bands, and IgG index) is mainly done to diagnose the cause of transverse myelitis. The main mimics include vascular myelopathy, metabolic myelopathy, compressive myelopathy, neoplasm, and radiation myelitis. Blood tests are used to rule out autoimmune and infectious causes. A high-dose intravenous corticosteroid (IV methylprednisolone 1 g/day for three to seven days) is the usual initial treatment started, failing which plasmapheresis is done next, although the sufficient evidence in support of the above two and other treatment options such as rituximab, azathioprine, cyclophosphamide, and IVIG is lacking [1,4].

The literature review regarding the anesthetic technique of choice in parturients diagnosed with transverse myelitis is limited and inconclusive. Some studies in literature such as those done by Lucas et al., Jha et al., and Seok et al. link the neuraxial technique as an attributing factor to transverse myelitis [6-8]. Providing general anesthesia to these patients using muscle relaxants for intubation is also problematic. Succinylcholine use for rapid sequence induction in these patients may cause severe hyperkalemia due to denervation and upregulated acetylcholine receptors [4,9]. The use of non-depolarizing muscle relaxant in this disease with denervated muscle may also cause a problem, as was the case seen in the study done by Weekes et al. where they used rocuronium only to find that the patient developed prolonged residual paralysis and was put on a ventilator postoperatively, which was later finally reversed by sugammadex [10].

A literature review produces one acceptable case report published by Saxena et al. in which a cesarean section was performed under monitored anesthesia care using ketamine in divided doses [5]. The patient in the study had complete sensation loss below the T8 dermatomal level, which supposedly would have decreased the need for analgesia.

The use of LMAs in pregnant patients is not a new concept now. Various studies have used supraglottic laryngeal mask airways in cesarean section. Halaseh et al. in a case series reported the experience of using ProSeal LMA in 3,000 elective cesarean sections [11]. Their study did not detect any evidence of aspiration. It concluded that ProSeal LMA might potentially obviate the pressor response to laryngoscopy and appears to be a good alternative to a tracheal tube for cesarean section. Saini et al. compared ProSeal LMA with endotracheal tube in 60 patients undergoing elective cesarean section and did not find any evidence of regurgitation with ProSeal LMA and therefore concluded that ProSeal LMA appears to be a safe alternative to endotracheal tube for cesarean section [12]. Han et al. in their research on 1,067 parturients used classic LMA and did not find any evidence of hypoxia, regurgitation, or aspiration [13]. These studies with a large sample size provide ample proof of the safety of supraglottic devices in pregnant patients [11,13].

With the available literature related to providing anesthesia in a parturient with transverse myelitis and the pre-anesthetic status of the indexed patient, keeping in view the emergency nature of the surgery, we decided to go ahead with general anesthesia with ProSeal LMA without using any curarization with a muscle relaxant. Propofol before delivery and fentanyl after delivery of a baby with sevoflurane and nitrous oxide combined with graded sensory loss below T6 worked well together. The use of the bispectral index helped prevent any intraoperative awareness. The patient was advised to follow up in the neurology department for further management.

## Conclusions

Providing anesthesia for an emergency cesarean section is always a challenge, significantly when pregnancy is associated with a neurological disease such as transverse myelitis, which comes with its challenges. We conclude that general anesthesia can be provided safely with ProSeal LMA without using muscle relaxants while taking necessary precautions in parturients with transverse myelitis. In these cases, we should always keep relevant emergency drugs in hand to deal with autonomic dysreflexia that can occur.
